# Rare Sequela of Spontaneous Coronary Artery Dissection

**DOI:** 10.7759/cureus.37645

**Published:** 2023-04-16

**Authors:** Muhammad Madkour, Ashis Mukherjee

**Affiliations:** 1 Cardiovascular Disease, Riverside Community Hospital, Riverside, USA; 2 Cardiovascular Disease, University of California Riverside School of Medicine, Riverside, USA

**Keywords:** acute coronary syndrome, flail mitral leaflet, ischemic mitral regurgitation, invasive coronary angiography, scad management

## Abstract

Non-atherosclerotic spontaneous coronary artery dissection (SCAD) is a rare cause of acute coronary syndrome. Here, we report a case of acute ischemic mitral regurgitation (MR) due to SCAD of the left main coronary artery. Given the extent of the acute ischemic MR and multi-vessel disease, the decision was made to pursue coronary artery bypass graft surgery and annuloplasty mitral valve ring repair.

## Introduction

Non-atherosclerotic spontaneous coronary artery dissection (SCAD) is a non-traumatic and non-iatrogenic separation of the coronary arterial wall and a rare cause of acute myocardial infarction. It is most commonly seen in younger women with no prior history of underlying coronary artery disease [1]. The overall incidence of SCAD is estimated to range from 0.28% to 1.1% based on angiographic assessments. In fact, the actual incidence is likely higher due to the substantial number of spontaneous dissections that present as sudden cardiac death [2]. The etiology is postulated to be multifactorial through a combination of underlying hormonal effects (peripartum period, multiparty), acquired arteriopathies, genetic factors, and systemic inflammatory diseases (Ehlers-Danlos, Marfan syndromes, and fibromuscular dysplasia) in conjunction with underlying stressors [3,4]. In three of the largest case series on SCAD, the percentage of women was found to be around 81-96%, with a mean age at diagnosis of 45-52 [5,6].

Based on angiographic characteristics, SCAD is classified according to the Saw classification and consists of three types [2,7]. Type I refers to the classic appearance of multiple arterial wall contrast staining. Type II (subdivided into Type IIA and IIB) is characterized by the presence of diffuse stenosis that can be of varying lengths. Type III mimics atherosclerosis as focal of tubular stenosis. A SCAD lesion on coronary angiography is identified by the pathognomonic manifestation of contrast dye staining of the arterial wall with the presence of dye hang-up [7]. As a result, subsequent coronary artery obstruction secondary to intimal disruption or intramural hematoma formation impedes coronary flow and myocardial injury transpires. As expected, the presentation of SCAD can be vast, ranging from angina pectoris, acute coronary syndromes, acute valvular disease, life-threatening ventricular arrhythmias, or even sudden cardiac death.

Even though SCAD is a rare cause of coronary pathology, this condition is associated with a high mortality rate. Early diagnosis of SCAD by coronary angiography is crucial because interventions such as urgent coronary artery bypass grafting (CABG) can be lifesaving. Here, we present a rare case of SCAD that resulted in acute valvular disease that required urgent CABG and surgical valve repair.

## Case presentation

A 53-year-old woman, gravida 6, para 4 with a history of mitral valve prolapse and hypertension presented with dyspnea and chest pain radiating to her jaw. The patient was previously in good health. Medications included hydrochlorothiazide and amlodipine. She denied any tobacco, alcohol, illicit drug use, or family history of early cardiac disease or cardiac-related deaths. The patient was post-menopausal and had four uncomplicated pregnancies. She had a previous workup with a transesophageal echocardiogram (TEE) that showed mild mitral regurgitation (MR) secondary to her mitral valve prolapse that involved the posterior leaflet.

On initial assessment, the patient was in mild acute distress. Vitals signs were stable. A cardiac examination revealed regular heart sounds and a grade III/VI holosystolic murmur best heard at the apex. No jugular venous distension or lower extremity edema was noted. Peak troponin I was 4.05 ng/mL (normal <0.08 ng/mL). Electrocardiogram showed ST depressions in the lateral leads and T-wave inversions in the anterolateral leads, and telemetry showed short runs of non-sustained ventricular tachycardia.

**Figure 1:**
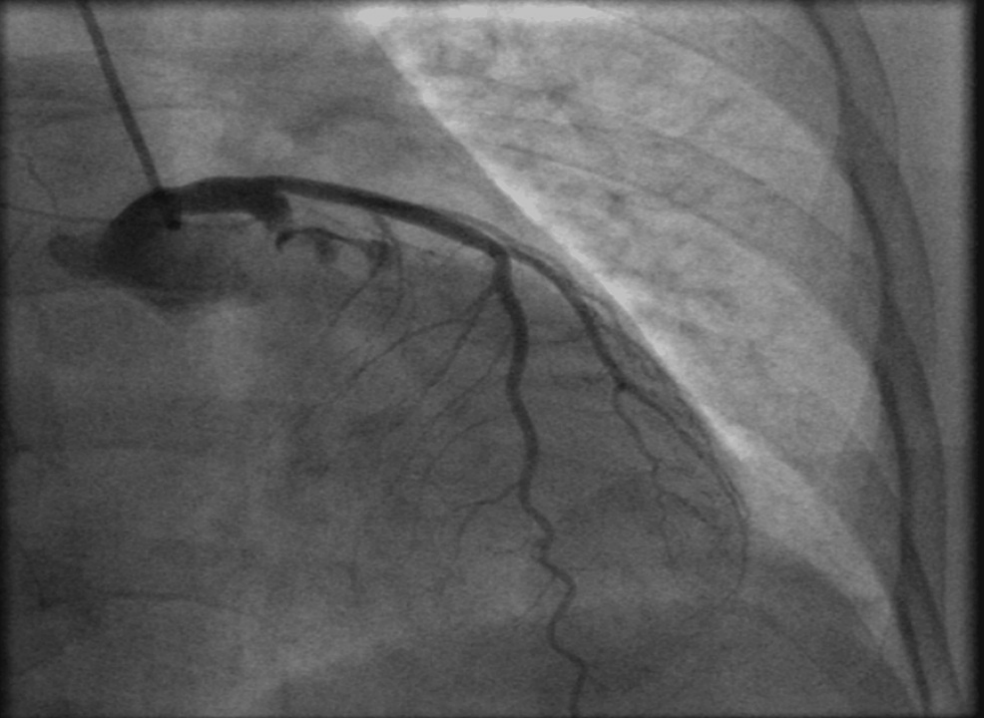
Left circumflex coronary artery with an ostial dissection tracking up to the left main coronary artery.

**Figure 2:**
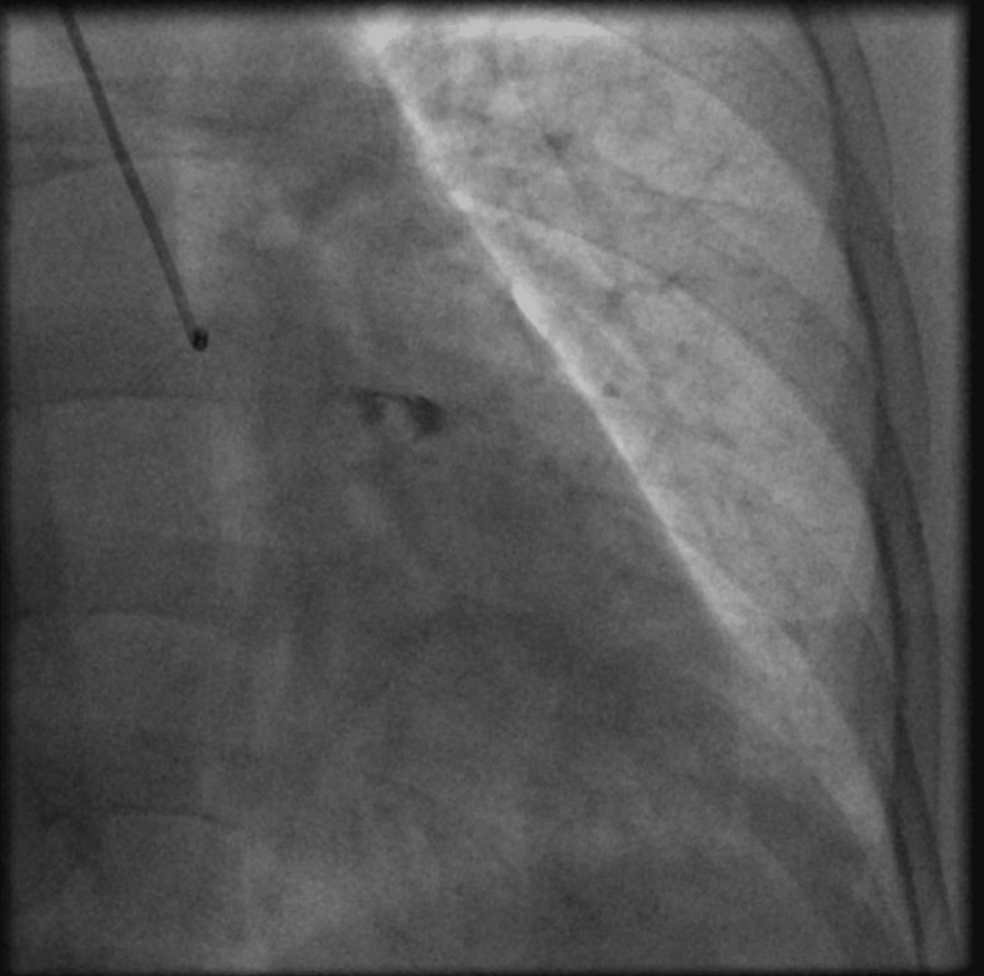
Still image of left coronary angiography showing dye hang-up in the left circumflex coronary system.

**Figure 3:**
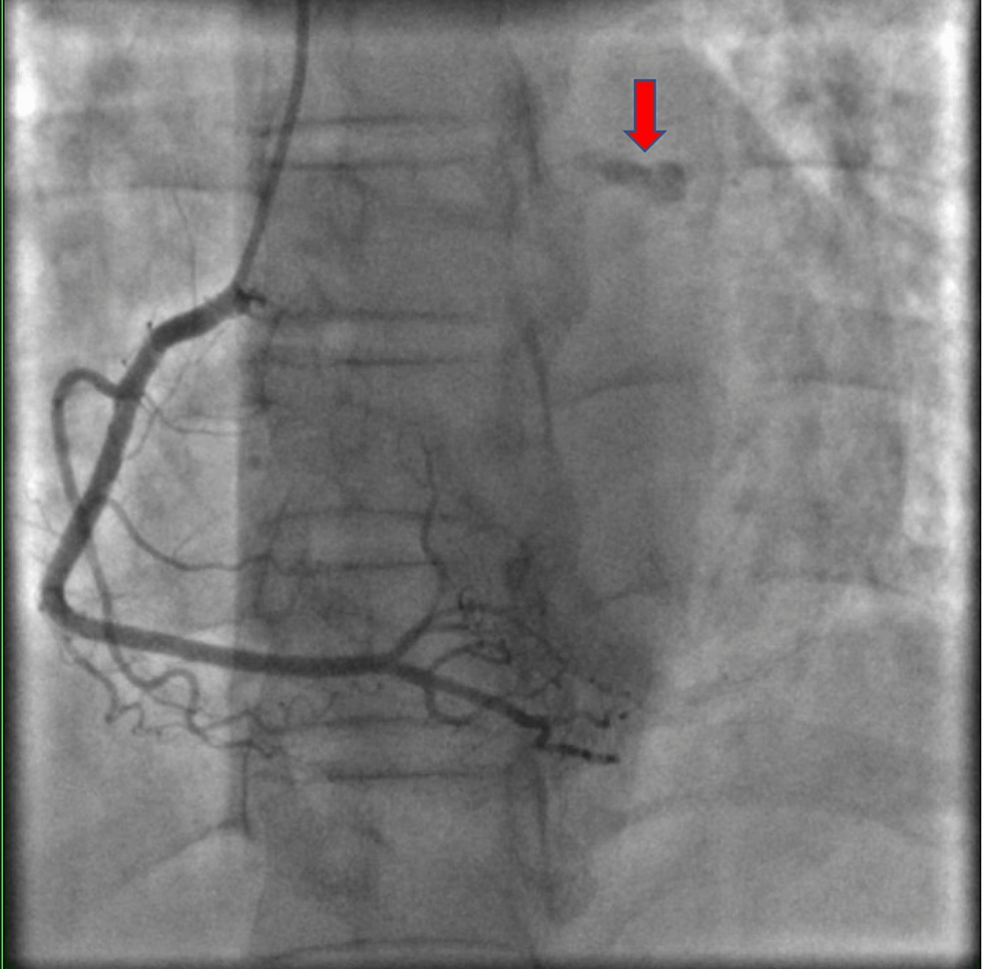
Selective right coronary angiography showing residual dye hang-up in the left circumflex coronary system (red arrow). The right coronary artery shows no significant coronary artery disease.

Cardiac catheterization with selective coronary angiography showed dissection of the left circumflex artery extending retrograde into the left main coronary artery with TIMI III flow (Video 1). There was a presence of dye hang-up in the left circumflex coronary artery seen with left and right coronary angiography (Figures 1-3).

**Video 1:** Selective left coronary angiography. Osital occlusion of the left circumflex coronary artery with residual dye hang-up, consistent with spontaneous coronary artery dissection.

**Figure 4:**
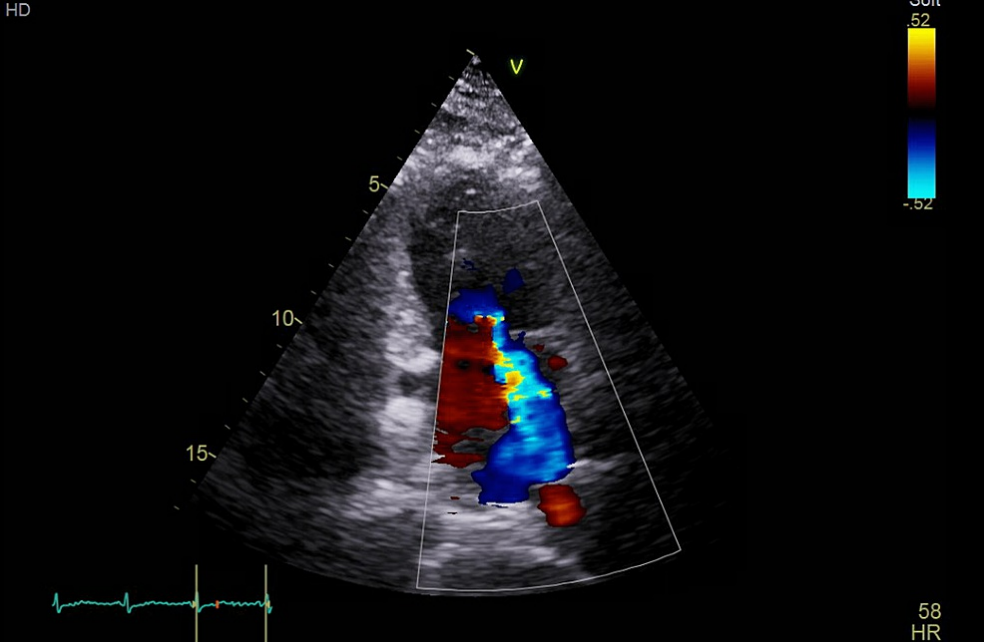
Four-chamber transthoracic echocardiogram showing severe mitral regurgitation.

Severe MR with an ejection fraction of 45% was noted by ventriculography and transthoracic echocardiography (Video 2, Figure 4). TEE confirmed a flail posterior mitral valve leaflet with a severe posteriorly directed regurgitant jet. Given the MR jet angle, this was likely associated with the tethering angle of the posterior mitral leaflet. This established the diagnosis of acute ischemic MR. Because of the extent of the dissection, she underwent urgent two-vessel CABG surgery with the left internal mammary artery to the left anterior descending artery and a reverse greater saphenous vein graft to obtuse marginal. In the context of the mitral valve being only mildly enlarged, mitral valve repair was performed via a trans-septal approach with a #30 Cosgrove Edwards annuloplasty band. Given that the mitral ischemic component of the MR was significant, the strategy employed was to increase the coaptation by decreasing the AP diameter without actually resecting much of the P2 leaflet for fear of foreshortening the posterior leaflet even more.

**Video 2:** Parasternal long-axis transthoracic echocardiogram showing a posteriorly directed mitral regurgitant jet.

Intraoperative TEE was performed and MR was noted to be drastically improved, with minimal trace regurgitation, and ventricular function was preserved/slightly improved. From the initial onset of symptoms on the presentation until revascularization with surgery, the total time elapsed was less than 72 hours. Postoperative troponin level 24 hours post-CABG was found to be 0.9 ng/mL. The patient recovered well and was later discharged home in good condition.

## Discussion

SCAD may present with clinical manifestations similar to patients with acute coronary syndrome. The presentation of SCAD depends on the flow-limiting severity of the dissection and ranges from asymptomatic to sudden cardiac death. In our case, the SCAD resulted in acute ischemic MR given the left circumflex coronary co-dominance, in which the involved artery supplied the posterior leaflet of the mitral valve. Despite the history of the compromised posterior mitral valve leaflet secondary to the prolapse, the ischemic MR resulted in an acute flail leaflet with a severe MR posterior jet. As mentioned earlier, given that the MR jet direction was posterior, the proposed mechanism was likely secondary to posterior mitral leaflet tethering.

As many dissections heal spontaneously in greater than 90% of cases, conservative management is the cornerstone of treatment [8]. However, as seen in our case, surgical interventions are sometimes necessary based on the site of the lesion that might result in concomitant acute mitral valve disease. Current recommendations for the overall decision to pursue medical management, percutaneous coronary intervention (PCI), or surgical revascularization is based primarily on the clinical presentation, the extent of dissection, and the amount of ischemic myocardium at risk [8]. PCI may be required in some specific situations such as ongoing ischemia or total vessel occlusion [2]. Furthermore, CABG may also be necessary in the context of ongoing multi-vessel ischemia, patients with left main SCAD, and associated acute valvular disease as seen in our case [2]. Through extensive literature review, the available data on the results of bypass grafting in SCAD is limited to small case series, and, in general, these series described good outcomes [2,9]. With the advancements in coronary angiography and optical coherence tomography, more cases of SCAD are being diagnosed, and additional studies would be beneficial to aid in the treatment recommendations moving forward.

## Conclusions

Although rare, SCAD is an important cause of acute coronary syndrome that occurs mainly in young women with minimal atherosclerotic risk factors. Due to the potentially life-threatening nature of SCAD, rapid diagnosis, interventions, and treatment is paramount. Cardiologists should be aware of the diagnostic and therapeutic challenges of this disease entity.
